# Secondary Operation Risk Assessment Method Integrating Graph Convolutional Networks and Semantic Embeddings

**DOI:** 10.3390/s25061934

**Published:** 2025-03-20

**Authors:** Pengyu Zhu, Youwei Li, Peidong Xu, Ping Li, Zhenbing Zhao, Gang Li

**Affiliations:** 1 State Grid Jiangsu Electric Power Co., Ltd., Huaian Power Supply Branch, Huaian 223000, China; zpy.nwfrozen@gmail.com (P.Z.); 13952382959@139.com (Y.L.); 2 State Grid Jiangsu Electric Power Co., Ltd., Wuxi Power Supply Branch, Wuxi 214200, China; luckyboy1986523@163.com; 3 School of Electrical and Electronic Engineering, North China Electric Power University, Baoding 071003, China; zhaozhenbing@ncepu.edu.cn; 4 Department of Computer, North China Electric Power University, Baoding 071003, China; lg_seek@ncepu.edu.cn

**Keywords:** graph convolutional network, knowledge graph, semantic search, risk assessment

## Abstract

In the power industry, secondary operation risk assessment is a critical step in ensuring operational safety. However, traditional assessment methods often rely on expert judgment, making it difficult to efficiently address the challenges posed by unstructured textual data and complex equipment relationships. To address this issue, this paper proposes a hybrid model that integrates graph convolutional networks (GCNs) with semantic embedding techniques. The model consists of two main components: the first constructs a domain-specific knowledge graph for the power industry and uses a GCN to extract structural information, while the second fine-tunes the RoBERTa pre-trained model to generate semantic embeddings for textual data. Finally, the model employs a hybrid similarity measurement mechanism that comprehensively considers both semantic and structural features, combining K-means clustering similarity search with a multi-node weighted evaluation method to achieve efficient and accurate risk assessment. The experimental results demonstrate that the proposed model significantly outperforms the traditional methods in key metrics, such as accuracy, recall, and F1 score, fully validating its practical application value in secondary operation scenarios within the power industry.

## 1. Introduction

With the continuous expansion of power networks, the level of intelligence in distribution networks has significantly improved, placing higher demands on their operational safety, stability, and maintenance capabilities [1]. To meet these requirements, the maintenance mode of distribution network automation devices is transitioning from traditional scheduled maintenance to condition-based maintenance. In this transition, precise risk assessment of secondary operations is particularly crucial. Secondary operations directly affect the stable operation of the power grid and the safety of personnel and property. Inadequate or incorrect risk assessment can lead to severe safety incidents, including large-scale blackouts. Thus, it serves not only as a key step in ensuring operational safety but also as a critical factor in enhancing operational efficiency [2].

In the power industry, secondary operations are a vital component of system maintenance, where their safety and efficiency are closely tied to grid stability [3]. However, as power systems grow increasingly complex, the difficulty of risk assessment for secondary operations has also escalated. Traditional risk assessment methods mainly rely on expert judgment, making them insufficient when dealing with large volumes of unstructured textual data and intricate equipment relationship information. Moreover, these traditional methods are often highly subjective and lack reproducibility. Different experts may arrive at significantly different evaluations for the same task, leading to inconsistent and incomparable results [4]. The limitations of expert experience may also overlook potential risk factors, compromising the comprehensiveness and accuracy of assessments. For example, secondary operations involve vast amounts of non-structured data, such as operating procedures, equipment manuals, and historical failure records. While these data contain valuable risk information, the traditional methods struggle to extract and utilize them effectively [5]. Furthermore, the intricate relationships between devices in power systems mean that a fault in one device can trigger cascading failures, which are often beyond the scope of traditional expert-based methods.

To address these challenges, this paper proposes a hybrid model that integrates a GCN with semantic embedding. This model efficiently processes unstructured textual data and uncovers complex relationships between devices, actions, and risk levels, offering a novel perspective and solution for secondary operation risk assessment. The main contributions of this paper include the following:

1. The proposed model innovatively combines semantic embeddings generated by RoBERTa with structural embeddings extracted by a GCN. Unlike traditional methods that focus on either semantic or structural information, this hybrid model leverages both to improve evaluation accuracy.
2. This paper constructs a structured knowledge graph for secondary operation scenarios in the power industry and utilizes a GCN to extract structural information from graph nodes, providing reliable support for risk assessment.
3. This paper employs the K-means clustering similarity search algorithm to significantly reduce computational complexity. Additionally, a multi-node weighted evaluation method is introduced to comprehensively consider the risk scores of multiple similar nodes, further enhancing the precision and reliability of the assessment results.
4. We evaluate the proposed model on power datasets. Compared to the other models, our model achieves the best results in terms of accuracy, recall, and F1 score. Furthermore, an ablation study evaluates the effectiveness of combining semantic and structural embeddings, as well as the similarity measurement mechanism.

This paper is divided into five main sections: Section 1 introduces the research background and current status of risk assessment for secondary operations in the power industry, highlighting the limitations of the traditional methods. Section 2 provides a critical review of the related work, summarizing the existing research and emphasizing the theoretical value of the innovations presented in this paper. Section 3 elaborates on the methodology, progressing from the macro framework to the technical details. Section 4 covers the experimental setup and results analysis, providing a comprehensive validation of the model’s effectiveness. Section 5 concludes the paper by summarizing the findings, identifying the potential of the technology, and suggesting directions for future research.

## 2. Related Work

In the field of secondary operation risk assessment, the existing research mainly focuses on traditional risk assessment methods, knowledge graph applications, and semantic embedding technology.

Traditional risk assessment methods usually rely on expert judgment and manual analysis. Although these methods can provide effective assessment results in some cases, they also have obvious limitations. For example, expert systems perform risk assessment by simulating expert decision-making processes, but they rely too much on predefined rules and expert knowledge bases and have difficulty coping with complex and dynamic real-world scenarios [6]. In addition, although the analytic hierarchy process (AHP) and fuzzy comprehensive evaluation methods are widely used, they are usually subjective and overly dependent on the experience and judgment of the assessor, which may lead to inconsistency and deviations in assessment results [7,8]. The inherent subjectivity of these methods and their lack of scalability further limit their application in complex and changing risk environments.

As a structured knowledge representation method, knowledge graphs have gained wide attention in risk assessment applications in recent years. By organizing domain knowledge into graphs, knowledge graphs can effectively capture the complex relationships between entities and provide strong support for risk assessment. However, the existing research on knowledge graphs in the power sector [9,10], especially the research on fault prediction and risk assessment, mainly focuses on the structure and operating status of power systems [11,12]. These studies often ignore the complexity associated with secondary operation risk assessment.

Semantic embedding technology can capture the semantic similarity between texts by converting text data into high-dimensional vector representations. In the field of natural language processing, pre-trained language models such as BERT [13], K-BERT [14], and RoBERTa [15,16] generate high-quality semantic embeddings by training on large-scale corpora. These technologies have achieved remarkable results in tasks such as text classification, sentiment analysis, and information retrieval. In risk assessment, semantic embedding technology is also used to process unstructured text data and extract semantic information related to risks [17,18]. However, these technologies often find it difficult to fully capture the detailed contextual relationships in professional fields such as power systems, especially in the complex context of specific fields.

A GCN [19] is a neural network model specially designed to process graph structured data, which can effectively process graph data. In the field of traffic risk assessment [20], GCNs have demonstrated excellent performance. For example, a model combining a GCN with LSTM can simulate spatiotemporal relationships and perform risk assessment on different behaviors of vehicles [21]. In addition, there are studies that combine a transformer and a GCN to perform collision risk assessment on vulnerable pedestrians [22]. Although the GCN performs well in processing graph data, its application in secondary operation risk assessment is still relatively scarce.

In summary, although traditional risk assessment methods, knowledge graph applications, and semantic embedding technology have made significant progress in their respective fields, they still face many challenges in dealing with the complex risk scenarios of power secondary operations. To address these challenges, this paper proposes a hybrid model based on graph convolutional networks and semantic embedding technology, which aims to improve the accuracy of risk assessment by integrating structural information and semantic information.

## 3. Methodology

### 3.1. Model Architecture

**Figure 1:**
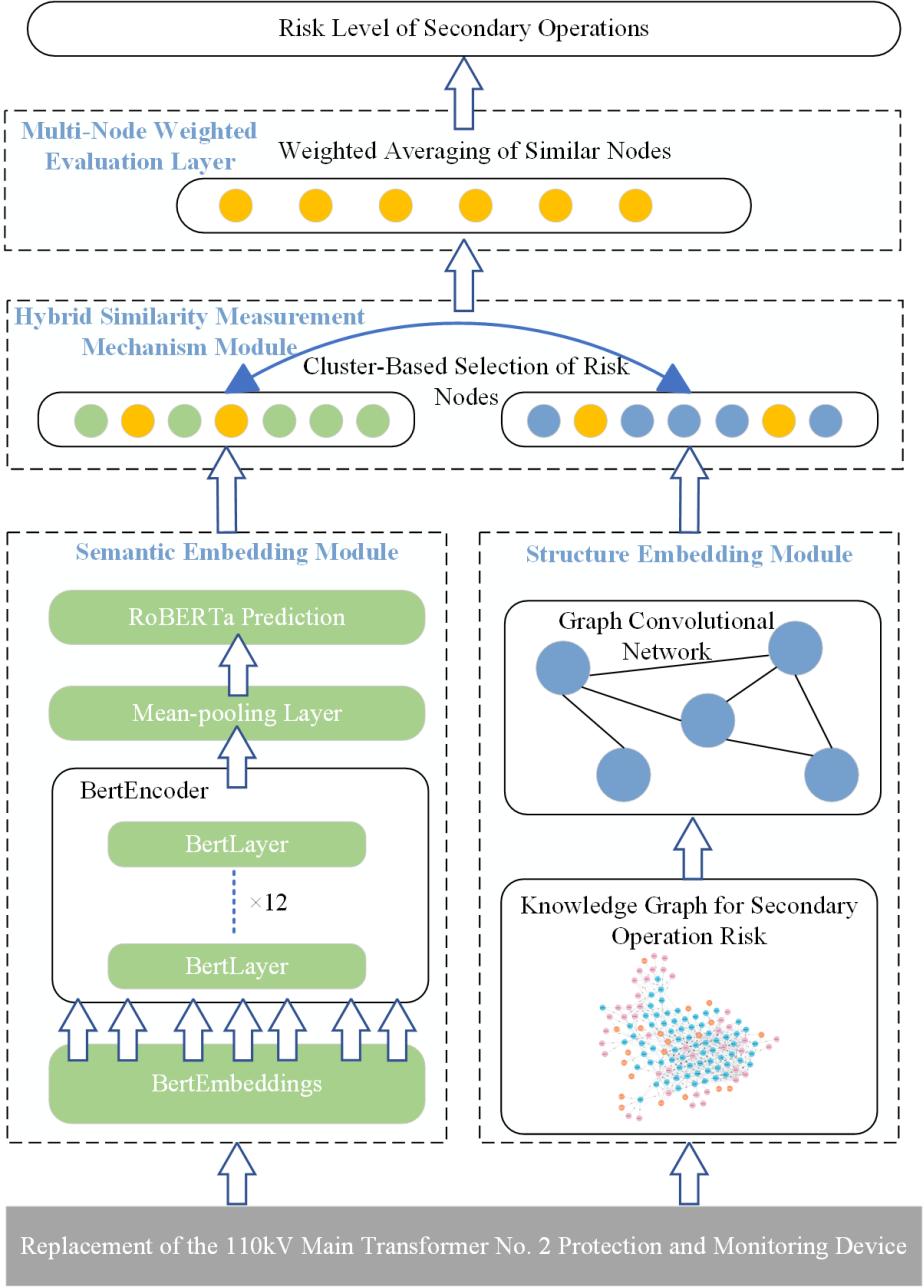
Overall framework of the model.

The overall framework is as shown in Figure 1, which mainly consists of two branches: semantic embedding branch and structural embedding branch. First, the input text generates deep semantic embedding through the RoBERTa model. After the feature dimension is compressed by the mean pooling layer, the context representation is strengthened by the 12-layer BERTEncoder to capture the semantic association. At the same time, the knowledge graph performs convolution operations through the graph convolutional network (GCN) to extract the structured representation of the nodes and capture the topological relationship between the nodes. Next, the hybrid similarity measurement mechanism is used to fuse the representations from the semantic embedding and structural embedding branches, and the K-means clustering method is used to screen out nodes with high similarity. Finally, the risk scores of similar nodes are aggregated through the multi-node weighted evaluation layer, and the risk level is finally output, thereby achieving accurate prediction and evaluation of secondary operation risks.

### 3.2. Knowledge Graph Construction

**Figure 2:**
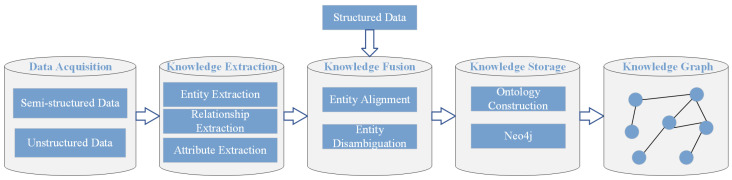
The construction process of the knowledge graph.

The knowledge graph for power secondary operations is specifically designed to assist personnel in efficiently conducting knowledge retrieval, precise risk classification, and informed decision-making during the secondary operation process. Through the meticulous construction of this knowledge graph, we are able to organize the professional knowledge, potential risk factors, and valuable empirical rules closely related to the operations in a structured manner, thereby providing robust assistance and support for on-site operations. The construction process of the knowledge graph is shown in Figure 2, In the figure, structured data, semi-structured data, and unstructured data represent the three main types of data sources. The knowledge extraction stage covers three core tasks: entity extraction, relationship extraction, and attribute extraction. The goal of entity extraction is to identify key entities in the text, relationship extraction is used to clarify the relationship between different entities, and attribute extraction focuses on capturing various attribute information of entities. In the knowledge fusion stage, there are two important tasks: entity alignment and entity disambiguation. Entity alignment aims to resolve entity conflicts between different datasets and ensure that multiple representations of the same entity can be effectively matched, while entity disambiguation is used to clarify ambiguous or unclear entity references to ensure the accuracy and consistency of knowledge. Finally, the knowledge storage stage involves ontology construction and the use of Neo4j. The main function of ontology construction is to define the hierarchical relationship between entities and their semantic rules. Neo4j is a structured storage system based on a graph database that can effectively store and query entities and their relationships.

#### 3.2.1. Data Acquisition and Ontology Construction

The data used for constructing the knowledge graph primarily come from the Electric Power Secondary Operations Risk Grading Table and the Relay Protection Risk Procedure Library. These materials provide comprehensive descriptions of the risk factors and operational standards involved in secondary operations in the electric power domain. During the data acquisition phase, this study employed various types of sensors to comprehensively monitor and collect risk factors associated with secondary operations in the power sector. The main sensors and their specific applications are as follows:

1. Current and Voltage Sensors: These sensors are used to monitor real-time changes in current and voltage on busbars, especially during retrofitting or testing processes. They can accurately capture fluctuations caused by line switching or equipment commissioning, providing critical data for early anomaly detection.
2. Fault Location Sensors: During equipment retrofitting, testing the response performance of protection devices may be necessary. Fault location sensors generate and capture fault signals to verify whether the protection devices are functioning correctly, ensuring system stability.
3. Environmental Monitoring Sensors: In scenarios where primary equipment remains in service, there may be heightened risks of electromagnetic interference and thermal effects. Environmental monitoring sensors, such as electromagnetic radiation sensors and temperature–humidity sensors, provide real-time surveillance of the working environment, assess its safety, and support risk mitigation efforts.
4. Grounding Resistance Sensors: These sensors are used to check whether the grounding status of out-of-service equipment meets safety standards. By accurately measuring grounding resistance, they help to prevent safety hazards caused by poor grounding.

The knowledge graph for secondary operations consists of two main levels: the ontology layer and the entity layer. The ontology layer defines the core concepts and their relationships within the domain, while the entity layer stores the specific entities and relationship information described in the ontology. This layered structure enables the systematic modeling of knowledge related to electric power secondary operations.

**Figure 3:**
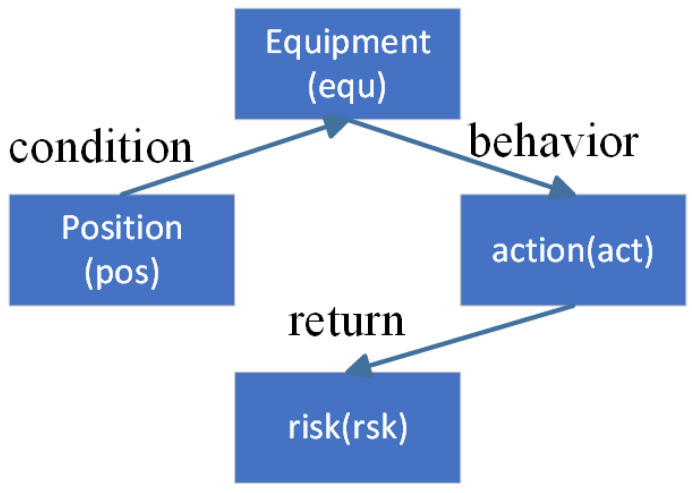
Ontology layer definition rules.

During the construction of the knowledge graph, domain experts in the electric power field provided guidance for the comprehensive summary of entities and relationships in the basic corpus, as well as the standardized definition of relationship types. The ontology layer’s definition rules are illustrated in Figure 3, covering four types of entities (position, equipment, act, and risk) and their relationships (condition, behavior, and return). This definition effectively describes the state and behavior of equipment under different conditions, along with the associated risk levels, thereby laying a solid foundation for subsequent text annotation and entity relationship extraction.

Since most existing electric power secondary operation texts are unstructured, preprocessing is essential before knowledge extraction. The preprocessing steps include the following:

1. Data Filtering: Cleaning the text corpus to remove irrelevant data and unnecessary characters that do not contain entity relationships, retaining only content relevant to knowledge extraction;
2. Sentence Segmentation: Splitting longer sentences into reasonable segments to ensure that each segment has an appropriate length and contains at least one entity relationship pair. This helps the model to better recognize entity relationships and improves extraction efficiency;
3. Data Annotation: The Label Studio annotation tool is used to mark entities in the text according to the BIO format. During this process, entities and relationships in the preprocessed text are precisely labeled, enabling the model to learn the structure and associations of domain-specific knowledge during training.

#### 3.2.2. Knowledge Extraction

Knowledge extraction is a core step in constructing the knowledge graph, responsible for identifying and extracting knowledge related to electric power secondary operations from preprocessed text. This stage comprises two main parts: named entity recognition (NER) and relationship extraction.

In the NER stage, the BERT-BiLSTM-CRF model is employed, consisting of three primary components. The base layer uses a bidirectional transformer as the encoder, fully capturing the associations between words and their contexts. BERT consists of 12 transformer layers, and the output *h* is obtained after processing through all 12 layers [23]. The computation in each transformer layer is as follows:

(1) $$G=\mathrm{LN}(H^{l-1}+\mathrm{MHAttn}\left(H^{l-1}\right))$$

(2) $$H^{l}=\mathrm{LN}\left(G+\mathrm{FFN}\left(G\right)\right)$$

where $H^{l}=\left\{h_{l}^{1},h_{l}^{2},...,h_{l}^{n}\right\}$ represents the output of the *l*-th layer, $H_{0}=E$; LN is layer normalization, MHAttn is the multi-head attention mechanism, and FFN is a feedforward neural network with ReLU as the activation function.

The output from the BERT layer is fed into the BiLSTM layer. At time *t*, given input $x_{t}$, the hidden layer output $h_{t}$ of the LSTM is computed as

(3) $$i_{t}=\sigma \left(W_{xi}x_{t}+W_{hi}h_{t-1}+W_{ci}c_{i-1}+bi\right)$$

where $W_{xi}$, $W_{hi}$, and $W_{ci}$ are weight matrices, bi is the bias, and σ is the activation function.

The Conditional Random Field (CRF) selects the label sequence with the highest probability as the prediction result [24]. The evaluation score of the CRF model is defined as

(4) $$s\left(x,y\right)=\sum W\left(y_{i}-1,y_{i}\right)+P\left(i,y_{i}\right)$$

where W is the transition matrix representing label transition scores, and $P(i,y_{i})$ denotes the score of the *i*-th character for its predicted label. CRF selects the optimal label sequence as the final prediction.

After completing NER, entity pairs are constructed from the text. For each entity pair, the sentence containing the pair is extracted as input for relationship extraction, and the relationship between the entities is annotated. In the relationship extraction stage, two separate multi-layer perceptrons (MLPs) are used to handle subject–object separation of input words. Basic MLP units are employed to extract features relevant to relationship classification. This feature dimensionality reduction process discards irrelevant information for relationship classification and retains first-order interaction features between subject and object words, directly representing the relationship between the two entities.

#### 3.2.3. Knowledge Fusion

Knowledge extraction provides entities, relationships, and attributes from unstructured and semi-structured data, forming the foundation for knowledge graph construction. However, due to the diverse and inconsistent quality of data sources, the extracted results often contain redundant and erroneous information. Further data cleaning and integration are required, involving two key steps: entity disambiguation and entity alignment. Entity disambiguation aims to address the issue of homonymous entities with different meanings in the extracted results. A CRF model is employed, leveraging defined feature and energy functions to establish relationships between observation and label sequences. The disambiguation task is achieved by maximizing conditional probability. The label prediction score is calculated as

(5) $$score\left(x,y\right)=\sum_{i=0}^{n}A_{y_{i},y_{i+1}}+\sum_{i=1}^{n}P_{i,y_{i}}$$

(6) $$P\left(y|X\right)=\frac{e^{s(X,y)}}{\sum_{f\in Y_{x}}e^{s(X,\bar{y})}}$$

where $P_{i,y_{i}}$ represents the emission score, which is the likelihood of the *i*-th character being predicted as the $y_{i}$ label. *A* denotes the transition score between labels. P(y∣X) indicates the probability of labeling the input sequence X as the sequence y. Entity alignment seeks to unify entities referring to the same object across different data sources. A logistic-regressionbased alignment model is utilized, expressed as

(7) $$P\left(y=1|x_{1},x_{2}\right)=\sigma \left(w^{T}x\right)$$

where $P(y=1|x_{1},x_{2})$ represents the probability of entity pair alignment under features $x_{1}$ and $x_{2}$. σ is the Sigmoid function mapping inputs to the [0, 1] range; *w* represents model weights, and *x* is the concatenated feature vector.

#### 3.2.4. Knowledge Storage

Neo4j is a user-friendly and extensible graph database, representing entities as nodes and relationships as edges. This graph-based structure facilitates intuitive visualization of custom knowledge graphs and enables efficient graph data operations through its powerful query language, Cypher. Users can employ Cypher statements to search, delete, and supplement relationships in the knowledge graph, significantly enhancing the efficiency of knowledge data management and utilization.

**Figure 4:**
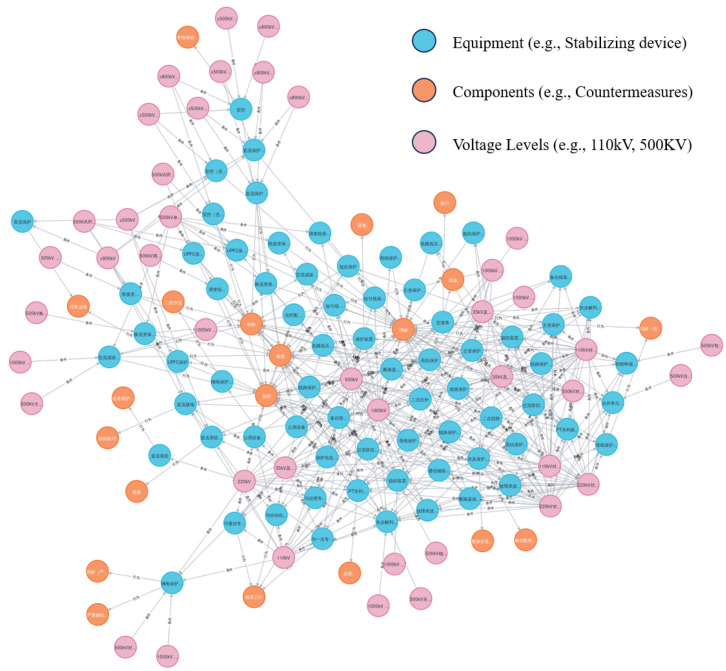
Sample of knowledge graph for secondary operations in electric power.

Moreover, Neo4j supports large-scale data processing, making it especially suitable for scenarios with multi-layered complex relationships. In the field of electric power secondary operations, the constructed knowledge graph effectively captures associations between equipment, processes, and risk factors. Figure 4 illustrates a segment of the knowledge graph, showcasing relationships between different nodes (e.g., equipment, actions, and risk levels). This visual structure helps users to better understand and manage such information.

### 3.3. GCN Structural Embedding Generation

**Figure 5:**
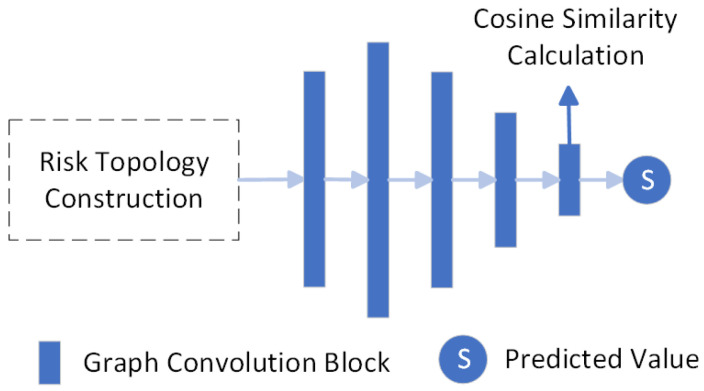
The schematic diagram of GCN architecture.

After establishing the operational knowledge graph in Neo4j, a GCN is subsequently used to extract its topological properties. A GCN is a neural network model specially designed for processing graph structured data. Its core idea is to update node representation by aggregating feature information of node neighbors. This feature enables the GCN to effectively model the topological associations between devices in the power system (such as the linkage relationship between relay protection devices and circuit breakers) and is particularly suitable for processing the propagation path analysis of the mutual influence of device states in secondary operations. As shown in Figure 5, the GCN structure designed for risk grading in secondary operations consists of four graph convolutional blocks and a cosine similarity computation unit. Each graph convolutional block contains three graph convolutional layers, one pooling layer, and one fully connected layer. Each block performs feature transformation and aggregation operations. The cosine similarity computation unit calculates the similarity between node features, supporting link prediction and node classification. Firstly, it is necessary to convert the structure of the knowledge graph into a format suitable for a GCN, which is the preparation stage of the graph structure. This means representing various entities in the secondary tasks as nodes in the graph and their relationships as edges. A graph G=(V,L) is defined, where *V* is the set of nodes ($V=E=E_{T}\cup E_{L}$) and *L* is the set of edges ($L=R=R_{P}\cup R_{L}$). In the context of secondary operation risk evaluation, the node set *V* includes position, equipment, act, and risk, while the edge set *L* consists of edges that describe the relationships among these nodes. Each node ν∈V is associated with a feature vector $f_{v}$. A feature matrix F is constructed, where each row $f_{v}$ represents the feature variables of a node *v*, including direct attributes and connectivity metrics derived from relationships such as degree and centrality. An adjacency matrix X is also constructed, where $X_{u\nu }=1$ indicates the presence of an edge between nodes u∈V and ν∈V, and $X_{u\nu }=0$ otherwise. For each node ν∈V, direct attributes are represented as a vector $A=\left\{a_{\nu_{1}},...,a_{\nu_{n}}\right\}$, and the in-degree and out-degree are calculated as follows:

(8) $$\deg_{\mathrm{out}}\left(\nu \right)=\left|\left\{u|\left(\nu ,u\right)\in E\right\}\right|$$

(9) $$\deg_{\mathrm{in}}\left(\nu \right)=|\left\{u|\left(u,\nu \right)\in E\right\}|$$

The in-degree centrality $C_{in}\left(v\right)$ and out-degree centrality $C_{\mathrm{out}}\left(\nu \right)$ are defined as

(10) $$C_{in}\left(v\right)=\frac{\deg_{in}\left(v\right)}{N-1}$$

(11) $$C_{\mathrm{out}}\left(\nu \right)=\frac{\deg_{\mathrm{out}}\left(\nu \right)}{N-1}$$

where *N* represents the total number of nodes. Therefore, the feature vector of each node *v* is expressed as $f_{\nu }=\left[a_{\nu_{1}},...,a_{\nu_{n}},\deg_{\mathrm{in}}\left(v\right),\deg_{\mathrm{out}}\left(v\right),C_{\mathrm{in}}\left(v\right),C_{\mathrm{out}}\left(v\right)\right]$

### 3.4. RoBERTa Semantic Embedding Fusion

This study uses RoBERTa, an improved version of the BERT model, as a pre-trained language model. RoBERTa uses a larger corpus for training and can effectively capture the semantics and contextual relationships in the text. By fine-tuning the dataset from the power sector, it can quickly converge and generate high-quality text representations in specific tasks, especially when dealing with texts containing professional terms and high contextual complexity.

At the initial stage of text processing, the input sentences are first tokenized into manageable lexical units. Additionally, special markers [CLS] and [SEP] are added to each input sequence: [CLS] is used for sentence representation (e.g., in classification tasks), while [SEP] is used to distinguish between sentences and paragraphs. These tokens are then transformed into word embedding vectors through an embedding layer, which maps each token into a fixed-dimensional vector space. These vectors not only capture the basic semantic meaning of the tokens but also retain subtle differences in meaning depending on the surrounding context.

To preserve positional information of tokens in the sentence, the model adds a positional encoding vector for each token. This vector uniquely identifies the position of a token in the sequence, enabling the model to understand the order of tokens. The vectors, enriched with semantic and positional information, are then fed into the transformer encoder for further processing.

The transformer encoder consists of multiple identical layers, each comprising two key components: self-attention mechanisms and feedforward neural networks. The selfattention mechanism allows the model to dynamically focus on other relevant tokens in the sequence while processing each token, thereby capturing long-distance dependencies. The feedforward neural network performs non-linear transformations on the attentionweighted vectors, further refining the semantic features. Through the iterative interaction of self-attention and feedforward networks across multiple layers, the model constructs increasingly rich and abstract semantic representations, laying a robust foundation for downstream tasks.

### 3.5. Similarity Measurement Mechanism

To accurately quantify the similarity between semantic embeddings and structural embeddings while improving the efficiency of similarity computation and the precision of similarity search, this study proposes a novel similarity measurement mechanism. Specifically, a hybrid similarity measurement method is designed to address the differences in representation between the text semantic embeddings generated by RoBERTa and the graph node embeddings generated by a GCN, enabling a more comprehensive capture of their relationships. Furthermore, by integrating the K-means clustering similarity search approach with a multi-node weighted evaluation strategy, the model achieves more precise risk assessments, ultimately delivering accurate and reliable risk level evaluation results.

The hybrid similarity measurement consists of two components: semantic similarity measurement and structural similarity measurement. Since both semantic embeddings and structural embeddings are represented as high-dimensional vectors, cosine similarity is used to evaluate the angular relationship between vectors, thereby calculating the semantic similarity. The formula is as follows:

(12) $$S_{sem}=\frac{h_{t}\cdot h_{n}}{\|h_{t}\left\|\right\|h_{n}\|}$$

where $h_{t}$ represents the text semantic embedding, $h_{n}$ represents the graph node embedding, $\|h_{t}\|$ and $\|h_{n}\|$ denote the norms of the two vectors, and $S_{sem}$ is the semantic similarity score. A score closer to 1 indicates higher similarity. This formula quantifies the similarity in the embedding space by calculating the angle between the two vectors. Additionally, a GCN learns the graph structure information in the risk knowledge graph of secondary operations to generate embeddings for each node. These embeddings incorporate not only the features of the nodes themselves but also the contextual information of their neighbors. Based on the local structural information of nodes, the structural similarity between a graph node embedding and its neighbors is calculated as follows:

(13) $$S_{str}=\frac{h_{n}\cdot h_{n}^{\prime }}{\|h_{n}\left\|\right\|h_{n}^{\prime }\|}$$

where $h_{n}$ represents the graph node embedding, $h_{n}^{\prime }$ denotes the neighbor node embedding, and $S_{str}$ is the structural similarity score. This approach captures the local structural information of graph nodes more effectively. Finally, semantic similarity scores and structural similarity scores are weighted and combined to obtain the final hybrid similarity score:

(14) $$S_{hsem}=\alpha S_{sem}+\left(1-\alpha \right)S_{sr}.$$

where $S_{hsem}$ is the hybrid similarity score, and α is the weight parameter used to balance semantic and structural contributions.

After similarity measurement, the next step is to use the hybrid similarity score to select the most relevant nodes as inputs for risk assessment. To improve the efficiency and accuracy of similarity search, clustering is performed on the embedding vectors prior to the search. Clustering reduces the search range in the embedding space and significantly lowers computational costs. Specifically, semantic embedding matrix $H_{t}$ and structural embedding matrix $H_{n}$ are constructed based on $h_{t}$ and $h_{n}$ respectively, and then concatenated to form the embedding matrix E. The K-means algorithm is applied to the embedding matrix [25]. Initially, *K* nodes are chosen as cluster centroids $C_{j}\left(j=1,2,...,K\right)$. For each sample $E_{i}$, its distance to each cluster centroid is computed, and the sample is assigned to the nearest cluster:

(15) $$\mathrm{Cluster}\left(E_{i}\right)=\arg\min_{i}{∥E_{i}-C_{j}∥}^{2}$$

Cluster centroids are updated based on the nodes within each cluster:

(16) $$C_{j}=\frac{1}{N_{j}}\sum_{E_{i}eC_{i}}E_{i}$$

where $N_{j}$ is the number of nodes in cluster *j*. After clustering, similarity calculation is performed on the embedding matrix. The similarity score is computed using cosine similarity:

(17) $$S_{i}=\frac{E_{i}\cdot h_{t}}{\|E_{i}\left\|\right\|h_{t}\|}$$

where $S_{i}$ is the similarity score between the node and the input embedding *h*. Similarity scores within the same cluster are ranked, and the top *K* nodes are returned as follows:

(18) $${\mathrm{TopK}}_{j}=\mathrm{argsort}\left(S_{i}\right)\left[-K_{k}:\right]\left[::-1\right]$$

where $K_{k}$ represents the number of nodes to return. After identifying the most relevant nodes in the secondary operation risk knowledge graph using the innovative similarity measurement and search methods, a multi-node weighted evaluation method is applied. This method calculates the final risk assessment by averaging the top K nodes’ risk values with their similarity scores as weights. The formula is as follows:

(19) $$L_{f}=\frac{\sum_{i=1}^{K}S_{i}\times L_{i}}{\sum_{i=1}^{K}S_{i}}$$

where *L* represents the final risk level, $S_{i}$ is the similarity score of the top K nodes, and $L_{i}$ is the risk assessment value of each node.

## 4. Results

### 4.1. Datasets and Parameter Settings

In order to quantitatively evaluate the proposed framework, this paper conducted a series of experiments based on actual power operation data. The dataset contains 3240 annotated work orders from the State Grid, combined with the knowledge graph in the power sector. These work orders cover multiple risk classification cases related to secondary operations, involving factors such as voltage level, high-risk operation content, and risk level of operation, etc. For details, see Table 1. In addition, the knowledge graph in the power sector is shown in Figure 4, which provides the relationship between equipment, operation, and risk, providing valuable structured knowledge for risk assessment.

In the data annotation process, this paper uses the Label Studio annotation tool and annotates the entities in the text according to the BIO format. The specific annotation form is shown in Table 2. The BIO format is a widely used annotation method for sequence annotation tasks, where “B” indicates the start position of the entity, “I” indicates the internal part of the entity, and “O” indicates the non-entity part. With this annotation method, the entities and their boundaries in the text can be accurately identified, and the type of entity can be determined, thereby providing high-quality annotation data for subsequent entity recognition tasks. Through BIO annotation, the entities and their boundaries in the text can be clearly identified, providing structured entity information for subsequent knowledge extraction tasks. This information is the basis for effective knowledge extraction. As a key component in the risk assessment model, knowledge extraction plays a vital role. In particular, correct knowledge extraction can identify the relationship between equipment and operations, providing valuable input for risk prediction. At the same time, the parameter settings of the model directly affect the quality of the subsequent model inputs, so an appropriate parameter configuration is crucial to improving the performance of the model. The specific parameter settings are shown in Table 3, which provides clear guidance for model training and evaluation.

### 4.2. Experimental Environment and Evaluation Metrics

The experiments in this paper were conducted on a computer with Ubuntu 18.04 LTS as the operating system, equipped with a GeForce GTX 2080 Ti GPU (Beijing Rongtianhuihai Technology Co., Beijing, China). The deep learning framework used is PyTorch 1.10, with Python version 3.7, and Cuda and CUDNN versions 10.2. The model learning rate was set to 1 × 10^5^, with 20 training epochs, and a batch size of 16 during training. The evaluation metrics of this model are precision (*P*), recall (*R*), and $F_{1}$ score. The formulas for these three evaluation metrics are as follows:

(20) $$P=\frac{T_{p}}{T_{p}+F_{p}}$$

(21) $$R=\frac{T_{p}}{T_{p}+F_{N}}$$

(22) $$F_{1}=\frac{2PR}{P+R}$$

where $T_{P}$ represents the correctly predicted instances, $F_{P}$ refers to instances that were predicted but incorrectly classified, and $F_{N}$ indicates the instances that were not predicted. The $F_{1}$ score is the harmonic mean of *P* and *R*, which provides a comprehensive reflection of the model’s predictive performance.

### 4.3. Results Analysis

In order to verify the performance of the knowledge extraction module, this study used the BERT-BiLSTM-CRF model for named entity recognition tasks and conducted comparative experiments with three baseline models. The detailed experimental results are shown in Table 4. The experimental results show that the BERT-BiLSTM-CRF entity recognition model significantly outperforms the other two baseline models. This advantage is mainly attributed to the powerful ability of the BERT pre-trained language model, which can dynamically generate word vectors, thereby significantly improving the performance of entity recognition. In addition, the BiLSTM model can more comprehensively extract contextual features by effectively capturing long-distance semantic information, further enhancing the model’s ability to recognize complex entities. Through this hybrid model combining BERT, BiLSTM, and CRF, this study successfully improved the accuracy and robustness of the named entity recognition task, fully demonstrating the unique advantages of the model in capturing semantic and contextual information.

In order to verify the performance of the proposed model in the classification of secondary operational risks, this paper conducted a comparative experiment to compare the proposed model with other existing models. The experimental results show that the proposed model achieved the best performance in terms of accuracy, recall, and F1 score. The detailed experimental results are shown in Table 5. From the experimental results, it can be seen that traditional methods such as Ranking SVM_EL and C-DSSM rely on artificial feature engineering or shallow networks, which have difficulty capturing the deep association of text semantics and the cascade risk between devices, resulting in strong subjectivity and weak generalization in the evaluation results. Although graph models such as GAT can effectively capture the topological relationship between devices, their ability to understand the semantics of pure text is limited, and they can only play a complementary role when combined with semantic embedding. Although pre-trained language models such as BERT learn contextual semantics through a large-scale corpus and significantly improve the ability of text feature extraction, they still lack sufficient relational reasoning ability. The model proposed in this paper combines the advantages of semantic understanding and relational reasoning and shows a more comprehensive performance in complex scenarios in the power field.

In order to verify the semantic embedding, structural embedding, and similarity measurement mechanism modules, this paper conducted a series of ablation experiments. The experimental results show that the model performs best when the three components exist at the same time. The detailed experimental results are shown in Table 6. When the semantic embedding is removed, the model’s text understanding ability is limited and it cannot fully capture the deep semantic information contained in the text description, resulting in decreases in both precision and recall and a decrease in F1 score. When the structural embedding is removed, it is difficult for the model to effectively model the relationship between nodes, resulting in a decrease in the ability to capture device cascade risks and ultimately causing a significant decline in the overall performance. When the similarity measurement mechanism is removed, the model lacks the means to effectively combine semantic and structural features, and it is difficult to achieve complementarity between different types of information, resulting in a decrease in model performance.

## 5. Conclusions

In this paper, a hybrid model integrating a graph convolutional network and semantic embedding is proposed to address the challenges of unstructured text processing and complex equipment relationship modeling in the risk assessment of secondary power operations. This method realizes the collaborative reasoning of equipment topological relationships and operation text semantics by constructing a knowledge graph in the power field. The GCN layer effectively captures the functional associations and risk transmission paths between secondary equipment. The dynamically fine-tuned RoBERTa model accurately analyzes the risk semantic features in the operation ticket text. The hybrid similarity measurement mechanism dynamically balances the contribution of semantic features and structural features through the α parameter, further improving the accuracy of the model. The experimental results show that the proposed model significantly outperforms the traditional methods in indicators such as precision, recall rate, and F1 score. In addition, the proposed model has great potential in a wider range of industrial applications. For example, in the field of smart manufacturing, it can assess the risks of automated production lines by analyzing the interdependencies between equipment and maintenance logs; in transportation systems, the model can combine infrastructure maps and maintenance records to assess the safety of railway signal operations. Since the framework can adapt to multi-source data, it has also become an important candidate technology for cross-industry risk management, and it is suitable for oil and gas pipeline monitoring, chemical plant safety assessment, and other fields. Future research will focus on three directions:

1. Building a dynamic knowledge graph update mechanism and using online learning to integrate new equipment parameters and operation and maintenance records in real time.
2. Developing a lightweight graph neural network architecture and adapting edge computing devices to achieve on-site risk assessment.
3. Exploring multi-agent collaborative evaluation models and optimizing the semantic embedding space in combination with expert experience feedback.

## Figures and Tables

**Table 1:** Sample of secondary operation risk classification.

No.	Voltage Level	High-Risk Operation Content	Risk Level
1	220 kV	Primary equipment area: Replacement of 220 kV terminal box, cable laying, and wiring; Replacement of 220 kV control panel, switchgear testing	III
2	35 kV or Below	Transformer row device modification	IV
3	110 kV	Main transformer protection: Control equipment replacement, voltage regulator modification	IV
4	1000 kV	First set of quasi-station equipment inspection for pressure control and circuit breaker	III

**Table 2:** Sample of BIO annotation for task content.

Token	Label
110 KV	B-pos
2nd	O
main transformer	B-equ
protection	I-equ
measurement	I-equ
installation	I-equ
replacement	B-act

**Table 3:** Knowledge extraction parameter settings.

Parameter Name	Parameter Value
Learning Rate	1 × 10^5^
Batch Size	8
Epoch	10
BERT Pre-trained Model	BERT-base-Chinese
BERT Dimension	768

**Table 4:** Results of entity recognition comparison experiments.

	P	R	F1
BiLSTM	0.766	0.801	0.785
BiLSTM-CRF	0.815	0.828	0.830
BERT-CRF	0.881	0.866	0.885
BERT-BiLSTM-CRF	0.898	0.885	0.890

**Table 5:** Results of comparison experiments.

Model	P	R	F1
Ranking SVM_EL	0.724	0.716	0.711
C-DSSM	0.807	0.816	0.808
BERT	0.811	0.825	0.822
RoBERTa	0.823	0.826	0.828
SBERT	0.834	0.837	0.834
ERNIE	0.843	0.841	0.839
GraphSAGE	0.763	0.755	0.760
GAT	0.845	0.838	0.841
Ours	0.879	0.863	0.868

**Table 6:** Results of ablation experiments.

Semantic Embedding	Structural Embedding	Similarity Measurement Mechanism	P	R	F1
✓			0.815	0.825	0.830
	✓		0.763	0.755	0.760
✓	✓		0.854	0.835	0.846
✓		✓	0.798	0.785	0.774
✓	✓	✓	0.879	0.863	0.868

## Data Availability

We apologize that the power data cannot be disclosed due to their particularity and confidentiality; further inquiries can be directed to the corresponding author.
